# Impact of embryonic *Fgf10* expression deficiency on embryonic mouse lung development and repair following hyperoxia injury

**DOI:** 10.1186/2194-7791-2-S1-A14

**Published:** 2015-07-01

**Authors:** CM Chao, D Al Alam, S Schermuly, H Ehrhardt, KP Zimmer, S Bellusci

**Affiliations:** Justus-Liebig-Universität, Gießen, Germany; Saban Research Institute, Los Angeles, CA USA; Excellence Cluster Cardio-Pulmonary System, Gießen, Germany

## Background

Bronchopulmonary dysplasia (BPD), a chronic lung disease of preterm infants, is characterized by impaired alveolar growth and pathologic vascularization.

## Aims

To investigate the impact of *Fgf10* expression deficiency on embryonic lung development and hyperoxia lung injury (BPD mouse model).

## Methods

1. Embryonic lungs (*Fgf10*^*+/-*^ and *Fgf10*^*+/+*^[WT]) were harvested at E12.5 and E18.5. Lung branches were quantified and H&E staining was performed.
2. After BrdU labeling lungs were harvest from embryos at E12.5. BrdU staining was performed on paraffin-embedded sections.
3. Transcriptomic analyses were performed by using whole lung RNA isolated at E18.5.
4. BPD mouse model:

*Fgf10*^*+/-*^ and *Fgf10*^*+/+*^*mice* were exposed to 85% O_2_ from P0-P8. Lung morphometric analysis, IHC staining (α-Actin/vWF staining, SPC, E-cadherin, Ki67, TUNEL), gene expression analysis (RNA isolated from type II alveolar epithelial cells [AEC II]), FACS (epithelial/ mesenchymal progenitor cells, AEC I/ II) were performed at P3.

## Results

1. Embryonic *Fgf10* heterozygous lungs exhibit epithelial branching defects and decreased Fibroblast growth factor signaling.
2. Embryonic *Fgf10*^*+/-*^ lungs show decreased epithelial proliferation.
3. Lungs of E18.5 *Fgf10*^*+/-*^ embryos display structural defects and abnormal gene expression.
4. *Fgf10* heterozygous pups display increased sensitivity to hyperoxia exposure associated with significant structural lung defects. Transcriptomic analyses show epithelial defects linked to cell cycle dysregulation and increased Tgfb signaling. *Fgf10* heterozygous vessels are more sensitive to hyperoxia injury and exhibit a less muscularized phenotype. SPC staining shows significant decrease in SPC+ cells after hyperoxia injury. FACS analysis revealed an increase of AEC I on the expenses of AEC II (progenitor cell).

## Conclusion

*Fgf10* deficiency leads to impaired embryonic lung development and death upon postnatal hyperoxia lung injury due to vulnerability of the epithelium.

